# Hetero-architectured core–shell NiMoO_4_@Ni_9_S_8_/MoS_2_ nanorods enabling high-performance supercapacitors

**DOI:** 10.1557/s43578-021-00318-y

**Published:** 2021-11-08

**Authors:** Lu Chen, Wenjing Deng, Zhi Chen, Xiaolei Wang

**Affiliations:** 1 Department of Chemical and Materials Engineering, Concordia University, 1455 De Maisonneuve Blvd. W., Montreal, QC H3G 1M8 Canada; 2 Department of Chemical and Materials Engineering, University of Alberta, 9211 – 116 Street NW., Edmonton, AB T6G 1H9 Canada; 3 Department of Building, Civil and Environmental Engineering, Concordia University, 1455 De Maisonneuve Blvd. W., Montreal, QC H3G 1M8 Canada

## Abstract

**Abstract:**

An effective technique for improving electrochemical efficiency is to rationally design hierarchical nanostructures that completely optimize the advantages of single components and establish an interfacial effect between structures. In this study, core–shell NiMoO_4_@Ni_9_S_8_/MoS_2_ hetero-structured nanorods are prepared via a facile hydrothermal process followed by a direct sulfurization. The resulting hierarchical architecture with outer Ni_9_S_8_/MoS_2_ nanoflakes shell on the inner NiMoO_4_ core offers plentiful active sites and ample charge transfer pathways in continuous heterointerfaces. Ascribing to the porous core–shell configuration and synergistic effect of bimetal sulfides, the obtained NiMoO_4_@Ni_9_S_8_/MoS_2_ as electrode material presents an unsurpassed specific capacity of 373.4 F g^−1^ at 10 A g^−1^ and remarkable cycling performance in the 6 M KOH electrolyte. This work delivers a rational method for designing highly efficient electrodes for supercapacitors, enlightening the road of exploring low-cost materials in the energy storage domain.

**Graphical Abstract:**

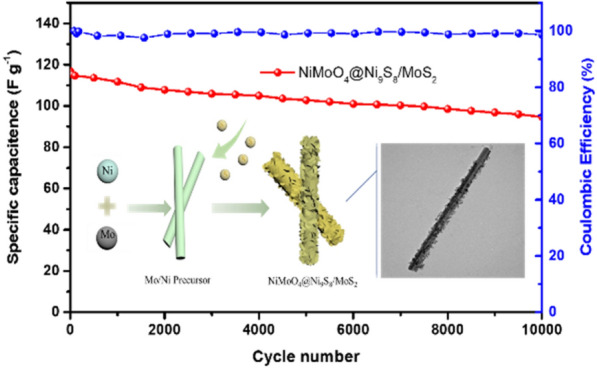

**Supplementary Information:**

The online version contains supplementary material available at 10.1557/s43578-021-00318-y.

## Introduction

Arising markets of high-energy density electronics and various electrical cars have prompted the production of efficient, cheap, and environmentally benign energy storage technologies and devices [1]. Among them, supercapacitors (SCs) have been attracted wide-ranging research interests because of their outstanding energy density, rating performance and superior cycling durability [2]. The SCs have filled the storage gap within traditional batteries devices and capacitors, so that they have been regarded as the promising and efficient storage devices. However, the energy density of supercapacitors still far below the Li-ion batteries which confines their large-print industrial employments [3]. Normally, supercapacitors are alienated to two types depending on different working principles: electrical double layer capacitors (EDLCs) and faradaic pseudocapacitors [4]. To boost the energy density and superior flexibility, asymmetric supercapacitors (ACS) consisted with the above two electrodes have aroused great interests, because they could be utilized the merits of both batteries and supercapacitors, and thus remarkably enhance their energy and power densities [5]. It’s known that carbon-based materials exhibit the excellent high conductivity and excellent density as battery-type electrodes. However, they still fail the expectation for long life durability. Compared to them, pseudo-capacitor materials possess rich Faradaic redox reactions active sites near the surface area of electrode, giving them excellent energy storage properties. Typical electrodes for pseudocapacitors are RuO_2_ and MnO_2_, but their usages are hindered because of their environmental harmfulness and low conductivity [6]. Recently, transition metal based materials, including oxides [6, 7], sulfides [8, 9] and hydroxides [10, 11] have become advanced pseudo-capacitor electrode materials. Because of their abundant oxidation states and multiple morphologies and structures, it’s favorable to facilitate the fast and reversible Faradaic redox reactions, thus resulting in high theoretical capacities. Particularly, NiMoO_4_ belonging to the binary oxide family is a good candidate because of its morphology diversity and outstanding capacitive efficiency [12]. Normally, NiMoO_4_ possesses two stable structures octahedral or tetrahedral configuration at low or high temperature, respectively [13]. Also, NiMoO_4_ is an unexpensive, plentiful, environmentally benign commodity and has significant nickel atomic capacity and high conductivity (10^–6^ S cm^−1^) from the molybdenum atom [14, 15]. For instance, Yun’s group published that gravel-like NiMoO_4_ grown on carbon cloth produced 970 F g^−1^ at 2.5 A g^−1^ [16]. Zhang, et al. synthesized hierarchical carbon sphere@NiMoO_4_ which displayed good specific capacity of 268.8 F g^−1^ at 1.0 A g^−1^ [17]. Interestingly, Wang’s group designed NiMoO_4_ with two morphology (nanospheres and nanorods), both of them showed the excellent specific capacitance, indicating NiMoO_4_ could be a promising candidate for supercapacitor [18]. Frustratingly, because of delayed kinetics reaction and intolerant morphology collapse during long charge/discharge procedure, NiMoO_4_ impedes low-rate capacity and poor cycling stability [19]. Even, it is still a big challenge to synthesize a NiMoO_4_ nanomaterials with large specific area and high electrical conductivity. So that not many reports are related to NiMoO_4_ electrode in application of supercapacitors. Consequently, structural modifications and constructions of NiMoO_4_ related to capacity and stability are of considerable importance to obtain advanced binary hybrids.

By contrast with metal oxides, transition metal sulfides (TMS) have higher conductivity, chemical durability or redox kinetics and are regarded as other potential pseudo-capacitance candidates [20]. Unlike oxygen, sulfur possesses comparatively low electronegativity, favoring for developing different nanostructures between metal ions and other species [5]. In particular, nickel sulfides have abundant redox active sites, forceful reducibility and inexpensive advantage exhibiting the excellent property for supercapacitor. For instance, Zhang’s group synthesized 3D flower-like nickel sulfide spheres with various morphologies exhibited initial discharge capacity of 550 mA h g^−1^ [21]. Osquian et al. reported three-dimensional starfish-like Ni_3_S_4_-NiS grown on graphene oxide with specific capacity of 1578 F g^−1^ at 0.5 A g^−1^ [22]. However, the cycle stability and rate performance of nickel sulfides still fail to reach the expectation. Wang’s group announced the provision of high capacitance three-dimensional Ni_3_S_4_ nanosheet electrodes but retained only around 60% capability after 2000 cycles [23]. However, another type of molybdenum disulfides (MoS_2_) displays superior cycling stability and pseudo-capacitance skills because of graphite-type layer structure and numerous valence states of Mo atoms, which can store charge between the different layers [24, 25]. In addition, atomic layers of MoS_2_ form a sandwich structure which molybdenum layer between two sulfur atomic layers bound together by Van der Waals forces favoring for ion intercalation and electron transfer [26]. Qi’s group synthesized hierarchical MoS_2_ nanospheres exhibited a specific capacitance of 142 F g^−1^at 0.59 A g^−1^ [27]. Kim et al. reported MoS_2_ spheres displayed capacitance retention of about 93.8% after 1000 cycles [28]. Unfortunately, chemically formulated MoS_2_ nanosheets are likely to aggregate, reducing surface area and leading to poor conductive ability. Hence, designing and developing of hetero-structured materials are necessary to enhance the conductivity, shortening the electron/ion transfer pathways and permit for high-rate capability.

One approach is to build hierarchical structures from multiple crystalline species that can effectively alternate electron configuration so that exhibit the superior electrochemical activity due to the synergistic effect of the heterostructure. A typical type of hierarchical structures is external structures which different porous structures such as nanowires, nanoparticles and nanosheets grown on the surface of the backbones such as nanorods or nanofibers owning higher specific surface current density and conductivity [29]. For example, hierarchical core–shell with strong interaction between the shell and core species greatly enhance the chemical and physical properties and offers the various interlayer [30]. Up to now, many contributions have been devoted in designing of the external hierarchical structures, consisting of metal sulfides with metal oxides. For instance, Jae Cheo’s group synthesized core–shell structural Co_3_O_4_@CdS showed 390 Fg^−1^ in symmetric supercapacitor device [31]. Tang et al. reported homogeneous core/shell NiMoO_4_-based nanowire arrayed on nickel foam showed amazing achievements with 47.2 Wh kg^−1^ at high energy density [32]. Hierarchical core–shell CoMoO_4_@NiMoO_4_ grown on nickel foam was synthesized by Zhang’s group, which showed a high capacity and excellent stability [33]. Qi et al. announced MnCo_2_S_4_@CoNi LDH core–shell heterostructures with high electrical conductivity and ample active sties offering better faradaic reactions as electrode for supercapacitor [34]. Therefore, construction of core–shell architecture is proved to be an effective strategy to utilize the merits of multiple components to the maximum to realize high-performance supercapacitors.

Herein, we develop a core–shell hierarchical architecture that core NiMoO_4_ nanorod is wrapped with Ni_9_S_8_/MoS_2_ nanoflakes, defined as NiMoO_4_@Ni_9_S_8_/MoS_2_, which is converted from the Mo/Ni precursor after sulfurization. This configuration offers mechanical protection and serves as the bridge connecting the outer metal sulfides and inner core. Due to the synergistic effect of its special multi-interfacial structure, fast ion/mass transportation in and ample active sites within 2D nanoflakes and 1D inner NiMoO_4_ nanorods, NiMoO_4_@Ni_9_S_8_/MoS_2_ nanorods demonstrate the excellent specific capacity of 373.4 F g^−1^ at 10 A g^−1^ and superior cycling property, implying that NiMoO_4_@Ni_9_S_8_/MoS_2_ can be a propitious electrode for high-efficiency energy storage system.

## Results and discussion

**Figure 1:**
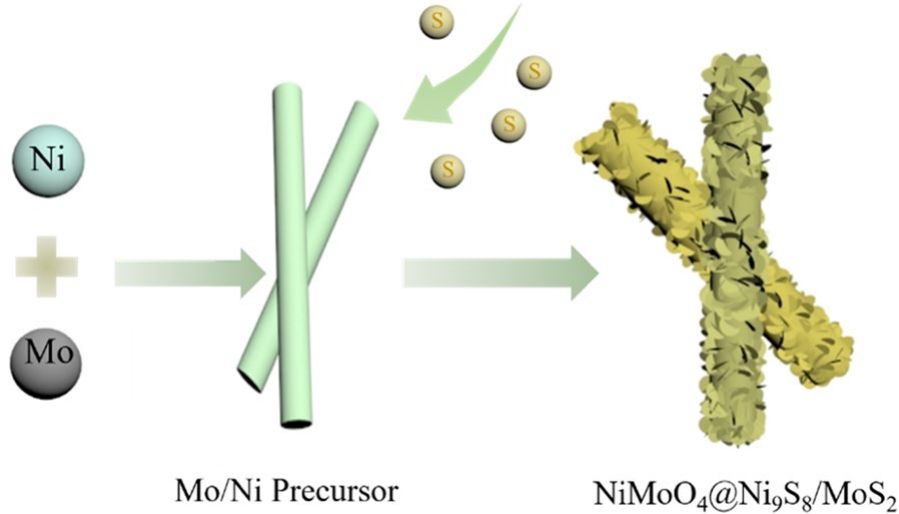
Scheme of the formation process of core–shell NiMoO_4_@Ni_9_S_8_/MoS_2_ hetero-structured nanorods.

**Figure 2:**
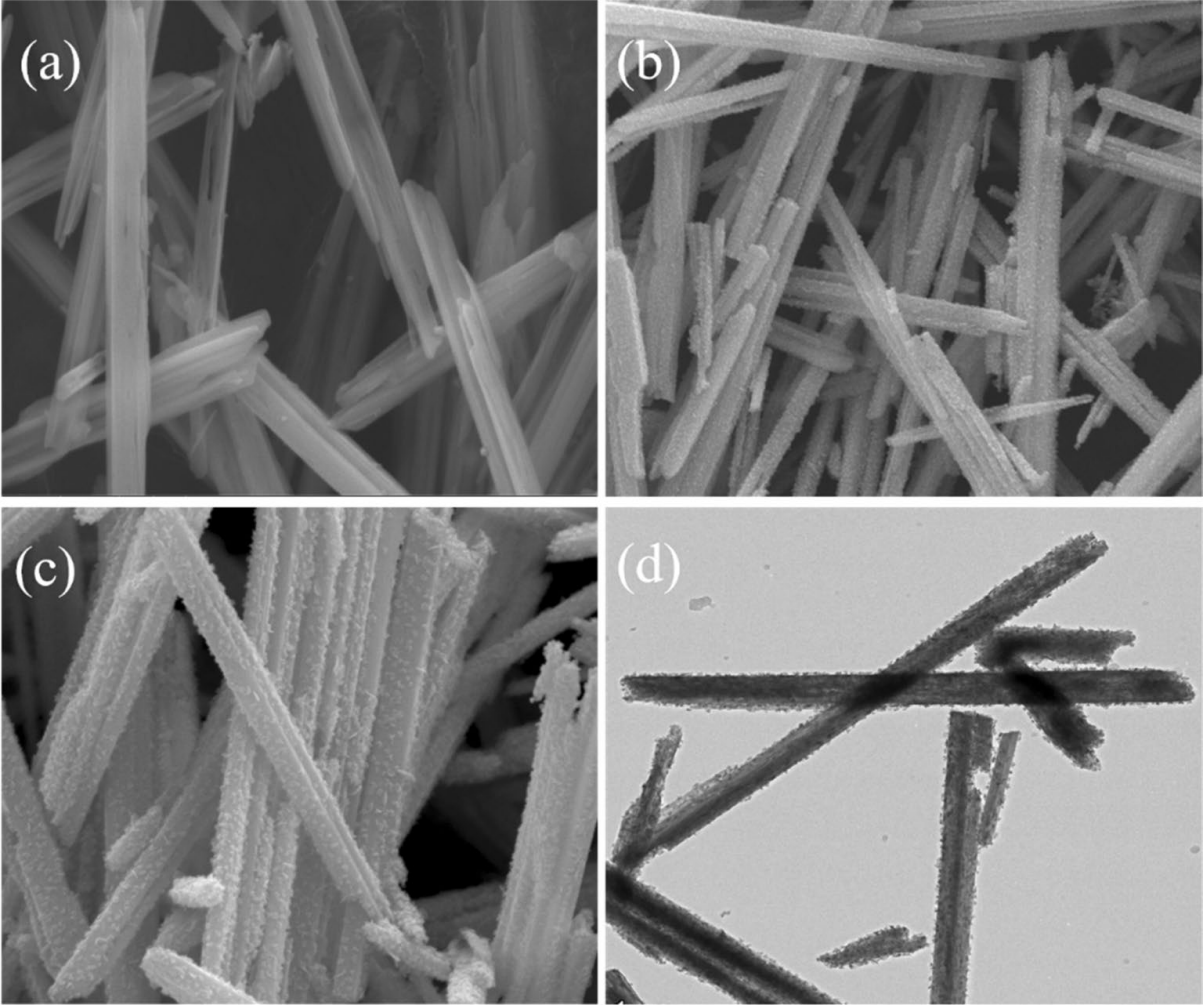
(a) SEM images of Mo/Ni precursor; (b-c) SEM images of NiMoO_4_@Ni_9_S_8_/MoS_2_ nanorods; (d) TEM image of NiMoO_4_@Ni_9_S_8_/MoS_2_ nanorods.

As illustrated in Fig. 1, the core–shell NiMoO_4_@Ni_9_S_8_/MoS_2_ heterostructure was converted from Mo/Ni precursor by a simple sulfurization method, as demonstrated in detail in the Materials and Method section. From the scanning electron microscope (SEM) image in Fig. 2a, it could be observed that Mo/Ni precursor possessed nanorod-like morphology with smooth exterior and a diameter of 130 nm. After the sulfurization method, the shell of Mo/Ni precursor converted to metal sulfides (Ni_9_S_8_/MoS_2_) with ultrathin nanoflakes shelling the inner core NiMoO_4_ to form a core–shell structure (Fig. 2b). By comparison, NiMoO_4_ nanorods without sulfurization remained the morphology with the smooth surface even some of the nanorods were broken, confirming that sulfurization could protect the nanorods from collapsing (Fig. S1). Figure 2c showed the clear structure and morphology of nanorods with a typical diameter of 180–200 nm with ultrathin nanoflakes and generated a hierarchical porous structure which further confirmed the formation of Ni_9_S_8_/MoS_2_. Such architecture was favorable for charge transfer and ion transportation to improve the electrochemical performance [35]. The detailed morphology of nanorods was discovered by transmission electron microscopy, which revealed that core–shell construction consisted of 2D Ni_9_S_8_/MoS_2_ nanoflakes as outer shell part and 1D nickel molybdate nanorods inner core (Fig. 2d). Coherent heterointerfaces could be observed in the entire nanorod between the outside metal sulfides and the inner metal oxide core, which could form the porous structure providing ample charge transfer channels.

**Figure 3:**
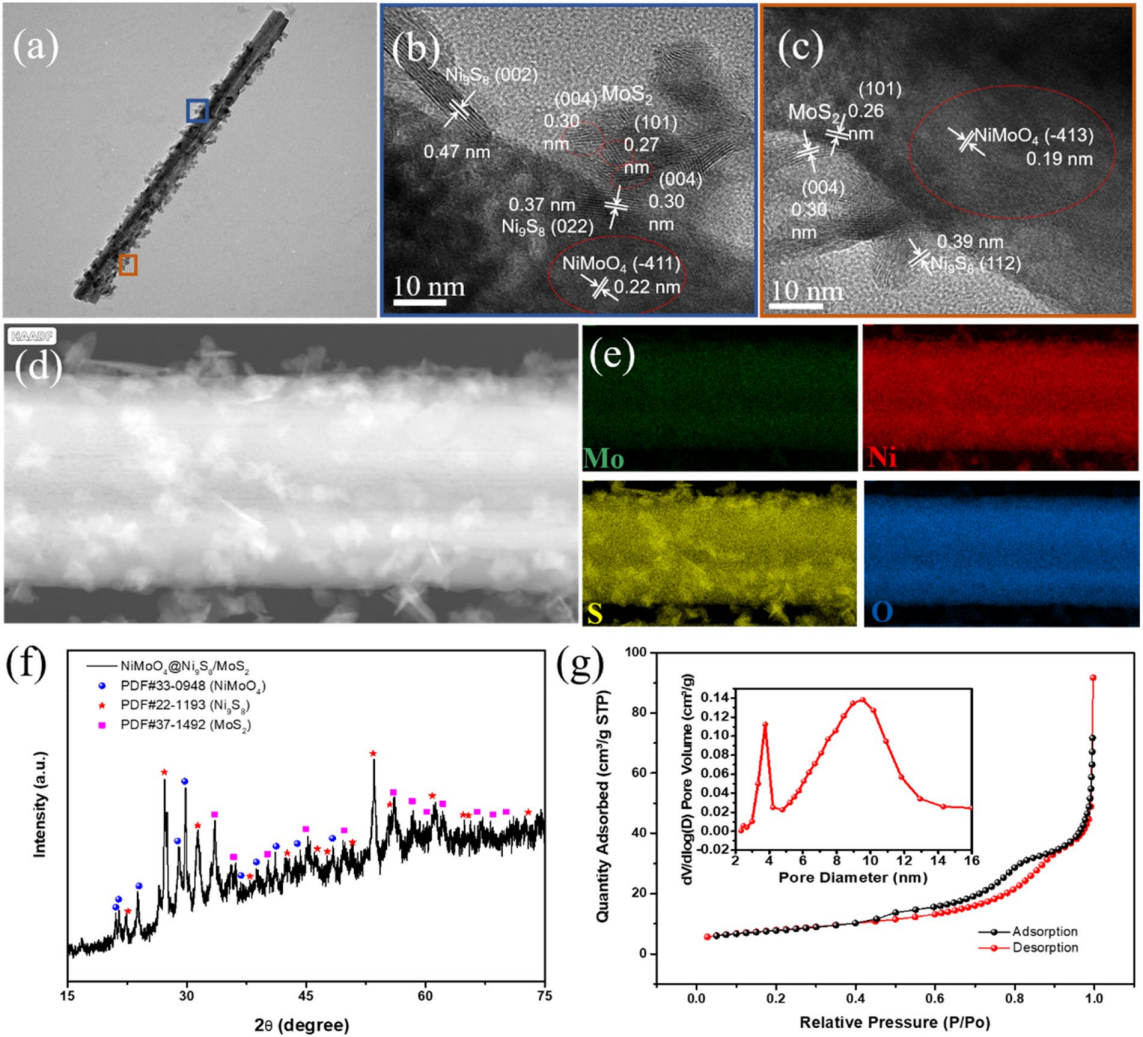
(a) TEM image of a single NiMoO_4_@Ni_9_S_8_/MoS_2_ nanorod; HRTEM images of (b) upper part (blue square) and (c) lower part (orange square) of NiMoO_4_@Ni_9_S_8_/MoS_2_ nanorod; (d) HAADF-STEM image of the selected part of NiMoO_4_@Ni_9_S_8_/MoS_2_ nanorod; and (e) the EDS element mapping spectra of element Mo, Ni, S and O; (f) XRD pattern and Rietveld refinement of NiMoO_4_@Ni_9_S_8_/MoS_2_; (g) N_2_ sorption isotherms of NiMoO_4_@Ni_9_S_8_/MoS_2_ (inset: pore size distribution profile).

The high-resolution TEM images of NiMoO_4_@Ni_9_S_8_/MoS_2_ in Fig. 3a-c which were selected from the upper (blue square) and lower part (orange square) from single nanorod. Figure 3b displays the crystalline spacings of 0.22 nm, 0.36 nm, and 0.27 nm, corresponding to (− 411) crystal lattice of NiMoO_4_, (022) crystal lattice of Ni_9_S_8_ and (101) lattice distance of MoS_2_, respectively, demonstrating ternary hybrids [36, 37] while Fig. 3c  demonstrates the existence of abundant interfaces between these components [38]. The uniformed distribution of Mo, Ni, S and O is shown in Fig. 3d-e, confirming the binary compound of Ni_9_S_8_/MoS_2_. It's worth mentioning that most Mo and O elements mainly dispersed in the core center, indicating that the incorporation of NiMoO_4_ inside the composite. XRD was investigated to depict the crystalline shapes of NiMoO_4_@Ni_9_S_8_/MoS_2_, as presented in Fig. 3f. The characteristic peaks at 23.9°, 28.8°, 29.7°, 41.2° and 47.4° confirmed the formation of NiMoO_4_ (JCPDS No. 33-0948) [39]. The five sharp peaks located at 27.2°, 31.3°, 42.6°, 50.8° and 53.4° were attributed to (202), (222), (332), (153) and (261) planes of Ni_9_S_8_ (JCPDS No. 22-1193) [40]. The typical peaks placed at 33.5°, 35.8°, 44.1°, 49.7°, 55.9° and 58.3° corresponded to the (101), (102), (006), (105), (106) and (110) planes of MoS_2_ (JCPDS No. 37-1492), respectively, reinforcing formation of ternary composites [41]. However, in Fig. S2a, Mo/Ni without sulfurization formed the nickel molybdate displayed as XRD pattern of NiMoO_4_. Figure 3g represents N_2_ sorption isotherms of NiMoO_4_@Ni_9_S_8_/MoS_2_ with VI-type curve of a hysteresis loop. The NiMoO_4_@Ni_9_S_8_/MoS_2_ surface area was 27.9643 m^2^ g^−1^, above the NiMoO4 (20.5 m^2^ g^−1^). Pore distribution profile in Fig. 3g and Fig. S2b demonstrated main product possessed pores at 0.6 nm, 3 nm and 10 nm, confirming the existence of micropores from the Ni_9_S_8_/MoS_2_ nanoflakes and mesopores from inner core. This porous structure of the main product could result from the introduction of sulfur during the calcination process under the high temperature, further creating more active sites and fastening electron transportation during the electrochemical procedure. The above result confirmed that the formation of metal sulfides and nickel molybdate with abundant pores that such porous structure could create more active sites and offer more charge transfer channels, shortening the ion diffusion distance, favoring electrochemical process.

**Figure 4:**
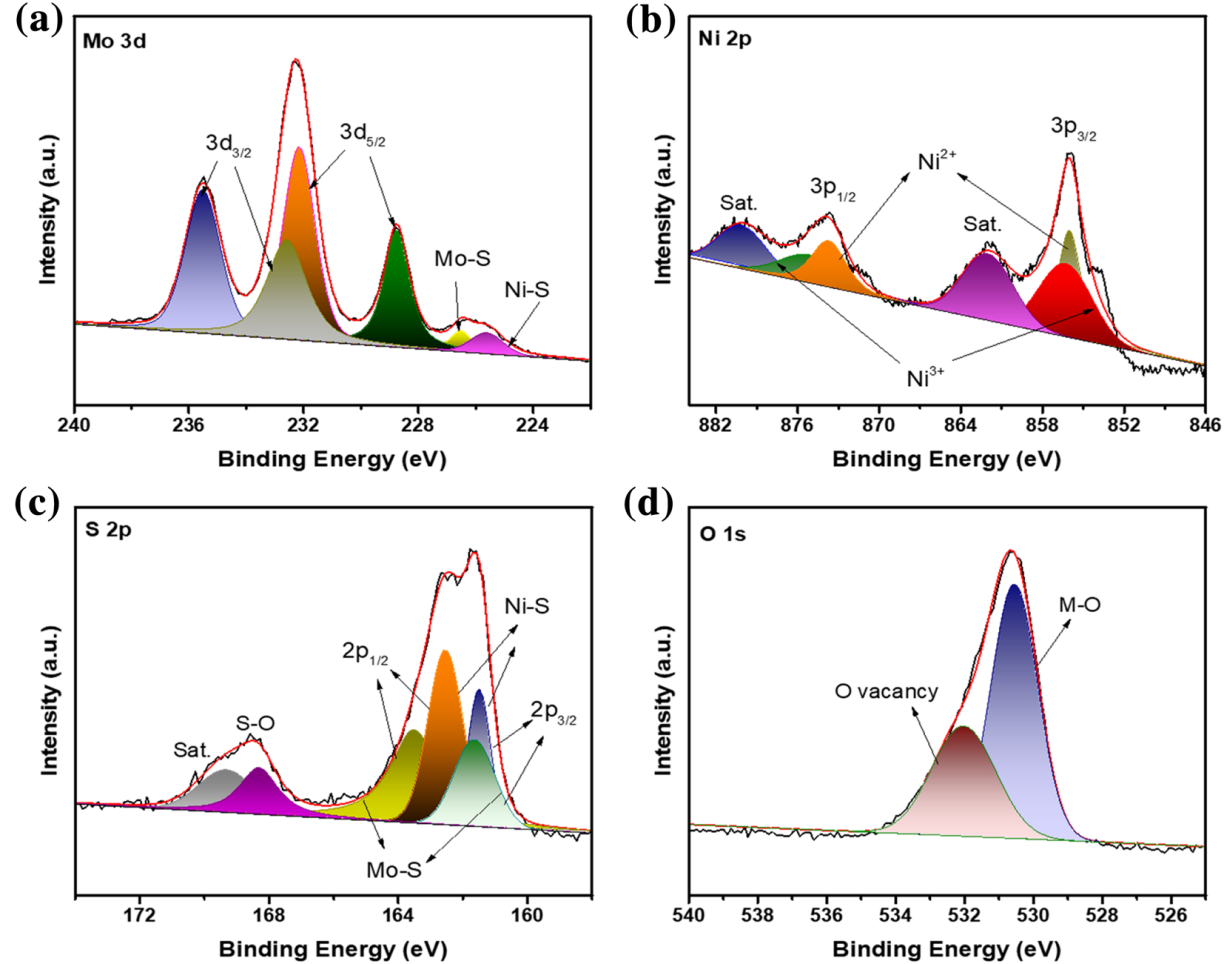
High-resolution XPS spectrum of (a) Mo 3d, (b) Ni 2p, (c) S 2p and (d) O 1 s of NiMoO_4_@Ni_9_S_8_/MoS_2_.

The element state analysis of the electrode was conducted through X-ray spectroscopy. In Fig. S3, XPS survey spectra revealed co-occur of Ni, Mo, S, and O elements in the nanorods. The Mo 3d XPS spectrum at the core stage could be divided into six peaks. (Fig. 4a). The two signals at 235.5 and 232.2 eV were accredited to Mo 3d_3/2_ and 3d_5/2_ of nickel molybdate, and signals at 232.6 and 228.7 eV corresponded to Mo 3d_3/2_ and 3d_5/2_ of molybdenum disulfide, respectively [42, 43]. The other two signals at binding energy 226.5 and 225.6 eV also agreed to S 2 s orbital of metal sulfide bond (Mo-S and Ni-S), respectively [44]. Figure 4b shows the Ni 2p spectra of two spin–orbit doublets with its satellites. Distinct signals placed at binding energy 855.9 and 873.7 eV were credited to 2p_3/2_ and 2p_1/2_ of nickel ion (II), and the other two peaks at 856.4 and 875.3 eV were associated with nickel ion (III) (2p_3/2_ and 2p_1/2_), respectively [45]. As presented of S 2p spectrum in Fig. 3c, the binding energy signals at 163.6, 161.5 eV were allocated with the 2p_1/2_ and 2p_3/2_ of nickel-sulfur bonding, two signals at 163.5 and 161.6 eV were assigned to the molybdenum-sulfur bonding (2p_1/2_ and 2p_3/2_), respectively [42, 46]. Also, a typical peak of the sulfur-oxygen bond with oxidation state appeared at signal 168.4 eV [47]. O1s spectrum is displayed as Fig. 4d, the signals at 530.6 and 531.9 eV corresponded to metal–oxygen (M–O) bond in nickel molybdate and oxygen vacancy of composites, respectively [19].

**Figure 5:**
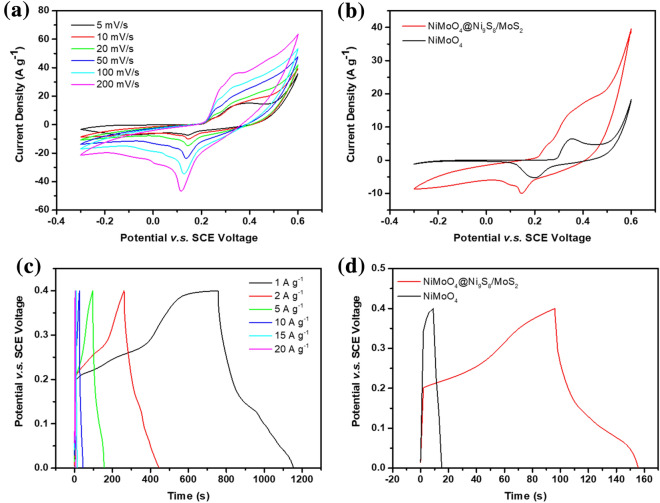
(a) CV curves of NiMoO_4_@Ni_9_S_8_/MoS_2_ at different scan rates. (b) CV curves of NiMoO_4_@Ni_9_S_8_/MoS_2_ and NiMoO_4_ at a scan rate of 5 mV s^−1^. (c) GCD plots of NiMoO_4_@Ni_9_S_8_/MoS_2_ nanorods at various current densities and (d) comparison of two electrodes at a current density of 10 A g^−1^.

The electrochemical outputs of all electrodes were delved by three-electrode configuration in alkaline solution. Figure 5a indicates CV curves of NiMoO_4_@Ni_9_S_8_/MoS_2_ from 5 to 200 mV s^−1^ in a voltage window of − 0.3 to 0.6 V (*vs.* SCE), respectively. Obviously, two redox summits were shown in a voltage window of 0.2–0.4 V and − 0.1 to 0 V, implying the typical valence state change of Ni^2+^/Ni^3+^ and faradaic redox between Mo^4+^ and Mo^6+^ during the electrochemical process [42]. Compared to the CV curves of NiMoO_4_ (Fig. S4a), the CV curves of the main product remained even at high 200 mV s^−1^, showing outstanding durability of NiMoO_4_@Ni_9_S_8_/MoS_2_. In addition, the sharp peaks slightly changed to a wide voltage window as the enlarging scan rate indicated fast faradaic reactions. It’s ascribed to the strong synergistic effect between Ni_9_S_8_/MoS_2_ and NiMoO_4_ due to the intimate hetero-interaction between them. It further verified the synthesis of metal sulfides hybrids and their typical pseudocapacitive behavior [24]. In Fig. 5b, CV plots of the main product and compared materials were illustrated to validate the merits of NiMoO_4_@Ni_9_S_8_/MoS_2_ nanorods for superior electrodes. Compared with NiMoO_4_ electrodes, the NiMoO_4_@Ni_9_S_8_/MoS_2_ nanorods had higher current densities and larger area, demonstrating a significant enhancement of capacitance due to multiple electron transport channels and synergistic effect within the metal sulfides and nickel molybdate [48]. Furthermore, the current density of the typical peak of NiMoO_4_@Ni_9_S_8_/MoS_2_ displayed a more linear and steep response than NiMoO_4_, suggesting larger surface area from the BET result, higher electrical conductivities, more active sites, and faster ion exchange due to the porous core–shell shape. Figure 5c displays the GCD plots of NiMoO_4_@Ni_9_S_8_/MoS_2_ nanorod with current densities ranging from 1 to 20 A g^−1^. Compared with NiMoO_4_ (Fig. S4b), NiMoO_4_@Ni_9_S_8_/MoS_2_ nanorod displayed a longer charge/discharge time of 446 s at 2 A g^−1^. The corresponding charge–discharge profiles of NiMoO_4_@Ni_9_S_8_/MoS_2_ and NiMoO_4_ were further provided in Fig. S4c and Fig. S4d. The obvious voltage plateau of NiMoO_4_@Ni_9_S_8_/MoS_2_ confirmed the “battery-type” pseudocapacitive behavior in comparison of NiMoO_4_ [49]. It’s reported that the bimetal precursor after sulfurization produced hierarchical Ni_9_S_8_/MoS_2_ nanoflakes with expanded interlayer spacing, which benefited to ion diffusion with enhanced capacitance [50]. The nonlinear shape of GCD plots further depicted the reversible pseudocapacitive properties which were mainly derived from the chemical conversion in NiMoO_4_, Ni_9_S_8_ and MoS_2_, which can be represented by the following equations [51, 52]:

1 $${\text{NiMoO}}_{{4}} + {\text{ OH}}^{ - } \leftrightarrow {\text{ NiMoO}}_{{4}} {\text{OH }} + {\text{ e}}^{ - }$$

2 $${\text{Ni}}_{{9}} {\text{S}}_{{8}} + {\text{ OH}}^{ - } \leftrightarrow {\text{ Ni}}_{{9}} {\text{S}}_{{8}} {\text{OH }} + {\text{ e}}^{ - }$$

3 $${\text{MoS}}_{{2}} + {\text{ K}}^{ + } + {\text{ e}}^{ - } \leftrightarrow {\text{ MoS}} - {\text{SK}}$$

Because of the sluggish potential reduction resulted from interior resistance and the faradaic chemical reaction, the discharge time decreased significantly as the current density increased [53]. More apparent results are presented in Fig. 5d. At 10 A g^−1^, the total charge/discharge duration of NiMoO_4_@Ni_9_S_8_/MoS_2_ composite was much superior to NiMoO_4_. Such excellent performance could be explained by more than one mechanism. First, the core–shell structure offered plentiful electron transportation pathways and functioned as a bridge connected the outer metal sulfides shell and inner NiMoO_4_ core, provided mechanical assistance to prevent the structure from collapsing and agglomerating during the faradic reaction, resulting in increased electrical conductivity. Second, the Ni_9_S_8_/MoS_2_ nanoflakes with abundant micropores and inner core NiMoO_4_ with mesopores possessed higher surface area and the heterogeneous increased electroactive sites for redox reaction enhanced the conductivity and prompted mass transport. Third, the synergistic effect between Ni_9_S_8_ and MoS_2_ contributed to high capacitance in the energy storage procedure.

**Figure 6:**
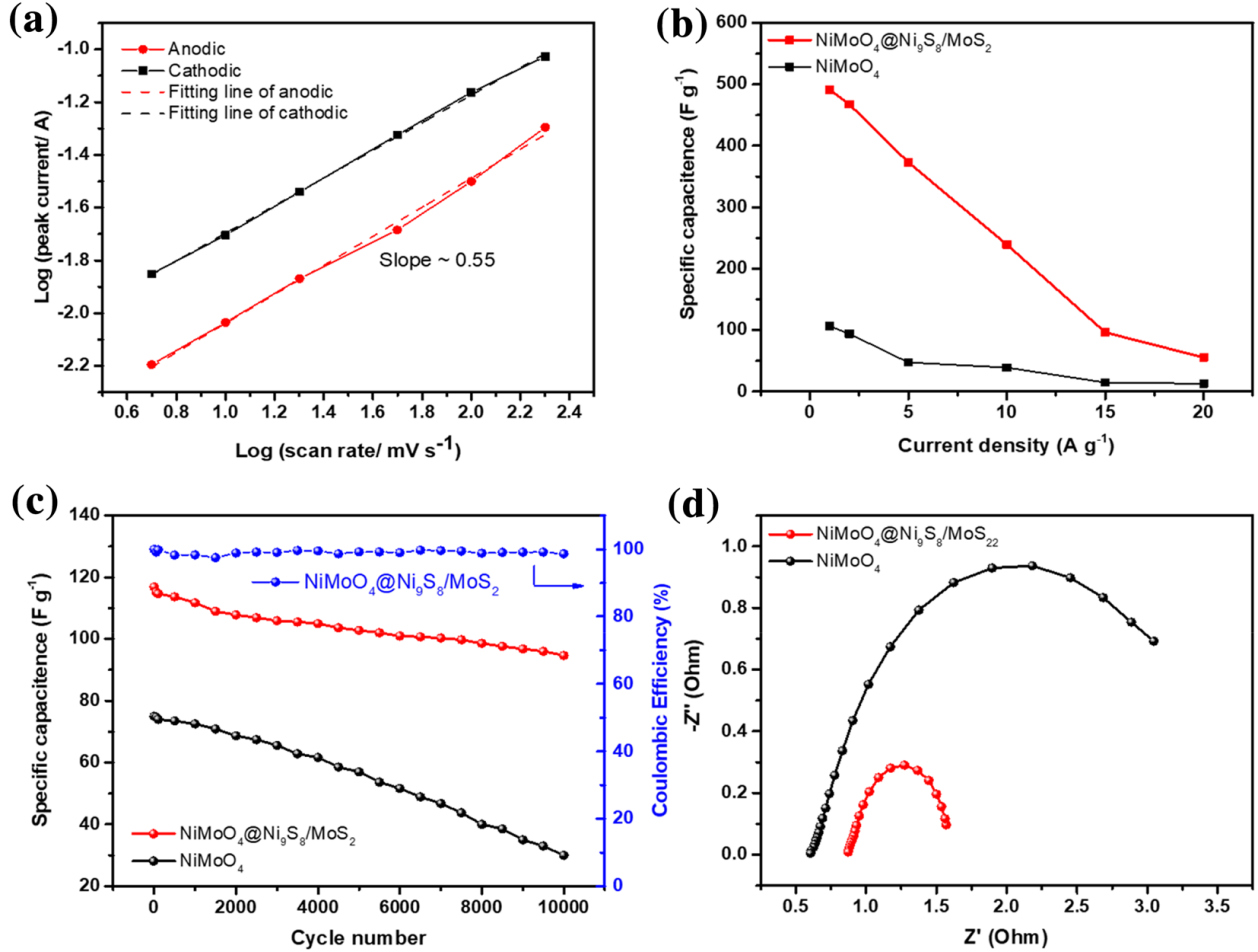
(a) Linear fitting plots of NiMoO_4_@Ni_9_S_8_/MoS_2_ for the log (peak current) and log (scan rate) for the cathodic and anodic peaks based on CV plots; (b) Specific capacitance of NiMoO_4_@Ni_9_S_8_/MoS_2_ and NiMoO_4_ at different current densities; (c) Cycling property and coulombic efficiency of NiMoO_4_@Ni_9_S_8_/MoS_2_ and NiMoO_4_ at a current density of 10 A g^−1^; (d) Comparison of Nyquist plots of NiMoO_4_@Ni_9_S_8_/MoS_2_ and NiMoO_4_.

Figure 6a exhibits the plot of log with peak current and log with scan speed at 5–200 mV·s^−1^ based on the CV plots of NiMoO_4_@Ni_9_S_8_/MoS_2_. Depending on the power-law [54]:

4 $${\text{i }} = \, \alpha \, \upsilon^{\beta }$$

where *α* was coefficient, *i* represented the maximum current, *υ* represented the scan rate. And *β *was the slope of the log (*i*) and log(v) plots. Accordingly, *β* = 0.5 indicated a diffusion-controlled process; *β* = 1 implied that the reaction was surface-controlled or had a capacitor-like feature [55]. Figure 6a shows that the value of *β* was close to 0.55, indicating that the redox process kinetics were determined by ion diffusion, further verifying the faradaic reaction in the NiMoO_4_@Ni_9_S_8_/MoS_2_ electrode. The special capacitances with different current densities depending on the GCD plot are presented in Fig. 6b. The specific capacitances of NiMoO_4_@Ni_9_S_8_/MoS_2_ electrode were 488.9, 467.6, 373.4, 240.1, 92.0, and 52.9 F g^−1^ at 1,2, 5, 10, 15, and 20 A g^−1^, respectively. By comparing with values of NiMoO_4_, it exhibited the superior rate performance. The cycling property of two electrodes is exhibited in Fig. 6c at 10 A g^−1^. After 10,000 cycles, the capacity retentiveness of the main product was 81.0% and coulombic efficiency remained almost at 100%, which is much better than NiMoO_4_ (40.1%) and other electrodes in Table S1, indicating its excellent cycling stability. The main reason of capacitance attenuation might be the difficulty of ions/electrons reaching to the thick area of material under 10 A g^−1^. However, the outstanding performance of NiMoO_4_@Ni_9_S_8_/MoS_2_ nanorods was ascribed to the core–shell structure which the outer metal sulfide shell protected the structure from collapsing and limiting fast electron transfer speed leading to the electrode decomposition, further confirming the merits of this architecture [13]. Figure 6d shows the typical EIS spectra of two electrodes before cycling. Obviously, the charge transfer resistance of the main product (0.7 Ω) was much smaller than the compared sample (2.41 Ω), which is correlated with electron transfer kinetics of redox reactions near the surface of electrolyte and electrode. According to the previous studies, such low charge transfer resistance was ascribed to plentiful electrochemical active sites and large cavities of micropores of the binary Ni_9_S_8_/MoS_2_ shell and interplayed with mass transport during the charge transport process [56]. It should also be mentioned that due to the lower electronegativity of sulfur than oxygen, bimetallic sulfides created a flexible space preventing the decomposition of structure caused by the elongation between atoms and made it easy for electrons to transport in the structure [57]. Consequently, after cycling, the resistance increasement of NiMoO_4_@Ni_9_S_8_/MoS_2_ from 0.7 Ω to 1.23 Ω (Fig. S4e). By comparison, NiMoO_4_ displayed the larger charge transfer resistance due to the collapsing structure in Fig. S4f. Depending on the above outcomes, the remarkable properties of NiMoO_4_@Ni_9_S_8_/MoS_2_ nanorods were credited to the subsequent explanations: (i) special core–shell architecture of nanorods protected the structure from collapsing and corrosion, serving as the bridge for outer metal sulfides and inner core leading to the high conductivity and supplied sufficient contact area between electrolyte and electrode; (ii) the ultrathin nanoflakes of Ni_9_S_8_/MoS_2_ provided more active sites and increased surface area and multiple micro/mesopores which provided the massive ion transport channels; (iii) the synergistic effect of typical heterostructure also favored the transport of ion and electrons.

## Conclusion

In conclusion, special core–shell NiMoO_4_@Ni_9_S_8_/MoS_2_ heterostructured was converted from Mo/Ni precursor by a facile hydrothermal process and followed by sulfurization, which worked as an efficient electrode for supercapacitor. It unveiled unsurpassed specific capacity of 373.4 F g^−1^ at 10 A g^−1^. Furthermore, the hybrid electrode remained excellent durability at 81.0% after 10,000 charge/discharge cycles. Such excellent electrochemical property was ascribed to special porous core–shell structure, increased surface area and ample active sites. This work demonstrated a facile method to design the hierarchical structure nanomaterials for supercapacitors.

## Materials and Methods

### Chemicals

Sodium molybdate dihydrate (Na_2_MoO_4_·2H_2_O), Nickel (II) nitrate hexahydrate (Ni(NO_3_)_2_·6H_2_O), Sulfur (S), Potassium hydroxide (KOH) were purchased from Sigma-Aldrich.

### Synthesis of Mo/Ni precursor

The conventional hydrothermal approach was used to prepare Mo/Ni precursors, followed by sulfurization. Sodium molybdate dihydrate (0.2 g) and nickel nitrate hexahydrate (0.2 g) were dissolved in deionized water (70 mL). Then the green transparent solution was moved to a 100 mL Telon autoclave and held at 180 °C for 12 h. The green final material was cleaned with deionized water and kept at 50 ℃ overnight, giving rise to Mo/Ni precursor.

### Synthesis of nanowire NiMoO_4_@Ni_9_S_8_/MoS_2_

Mo/Ni precursor and sulfur (mass ratio = 1:3) were put in one porcelain boat with the downstream and upstream side and calcinated at 350℃ for 2 h under flowing Ar. The obtained material was NiMoO_4_@Ni_9_S_8_/MoS_2_. For comparison, NiMoO_4_ was also synthesized by calcining Mo/Ni precursor without sulfur in the same calcination process.

### Characterization

FEI Quanta 450 Environmental Scanning Electron Microscope was utilized to examine the anatomy of materials. Transmission electron microscopy (TEM, Thermo Scientific Talos F200X G2 S/TEM) was tested to examine the crystal details of the materials. X-ray diffraction (XRD) measurements were used by a monochromatic Bruker D8 advance diffractometer. The composition and valence states were performed on a Thermo Scientific X-ray photoelectron spectroscopy. N_2_ sorption measurements were utilized using Micromeritics 3Flex Surface Characterization Analyzer with N_2_ as the adsorbent.

### Electrochemical Measurements

All electrocatalytic tests were conducted by Biologic VMP300 electrochemical workstation in 6.0 M KOH with three-electrode equipment including the glassy carbon (GC) electrode (area: 0.196 cm^2^) as the working electrode, platinum wire as the counter electrode and the saturated calomel electrode (SCE) as the reference electrode. Working electrode was prepared by adding the active nanomaterial and super P in 600 µL of 10% polyvinylidene difluoride (PVDF) dissolved in N-methyl pyrrolidinone (NMP). Then slurry (120 µL) was spread on a clean nickel foam (1 $\times$ 1 cm^2^) by drop casting and dried overnight. The dried electrode was then pressed using a hydraulic press at a pressure of 10 MPa. The mass loading of active material in single nickel foam was about 2 mg. The CV and GCD were determined within a possible window of − 0.3 to 0.6 V, and the material's specific capacity was estimated using GCD curves. The specific capacitance, *C*_sc_ (F g^−1^), were measured by the equation:

$${{C}}_{\text{sc}}= \frac{{I \times \Delta t}}{{m \times \Delta V}}$$

where *I* (*A*) was the discharge current, Δ*t* (s) represented the discharge time, Δ*V* (V) was the potential window, and *m* (g) was the mass of the active materials.

## Supplementary Information

Below is the link to the electronic supplementary material.

Supplementary file 1 (DOCX 617 kb)

## Declarations

### Availability of Data and Material

Data will be made available upon reasonable request.
