# Supplementary material for: Hetero-architectured core–shell NiMoO4@Ni9S8/MoS2 nanorods enabling high-performance supercapacitors
Source: J Mater Res. 2021 Nov 8;37(1):284–93. doi: 10.1557/s43578-021-00318-y (PMC8810477; doi:10.1557/s43578-021-00318-y)
Supplement: Supplementary file 1 — (DOCX 617 kb) [file 43578_2021_318_MOESM1_ESM.docx]

Supporting Information for

**Hetero-Architectured Core-shell NiMoO_4_@Ni_9_S_8_/MoS_2_ Nanorods Enabling High-Performance Supercapacitors**

*Lu Chen,^1^ Wenjing Deng,^2^ Zhi Chen,^3^* and Xiaolei Wang^1,2^**

^1^ Department of Chemical and Materials Engineering, Concordia University, 1455 De Maisonneuve Blvd. W., Montreal, Quebec H3G 1M8, Canada.

^2^ Department of Chemical and Materials Engineering, University of Alberta, 9211 – 116 Street NW., Edmonton, Alberta T6G 1H9, Canada.

^3^ Department of Building, Civil and Environmental Engineering, Concordia University, 1455 De Maisonneuve Blvd. W., Montreal, Quebec H3G 1M8, Canada.

**
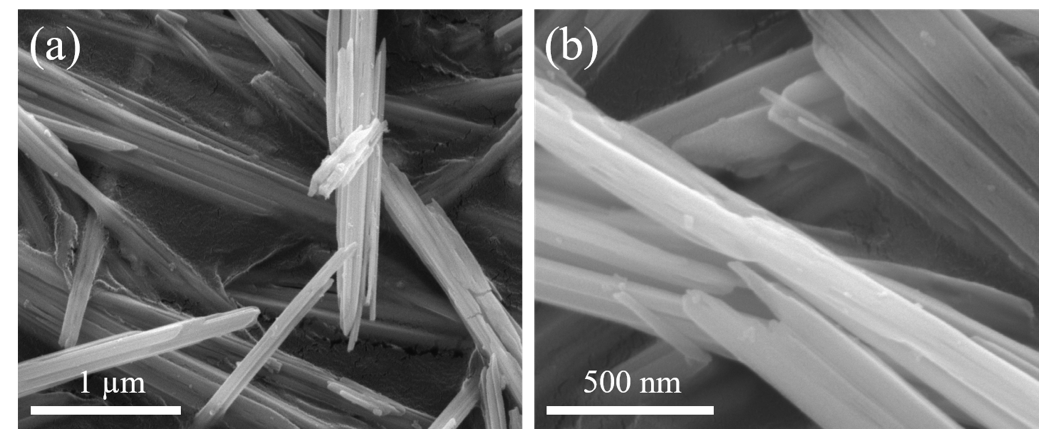
**

**Figure S1.** (a) SEM images of NiMoO_4_ nanorods.

**
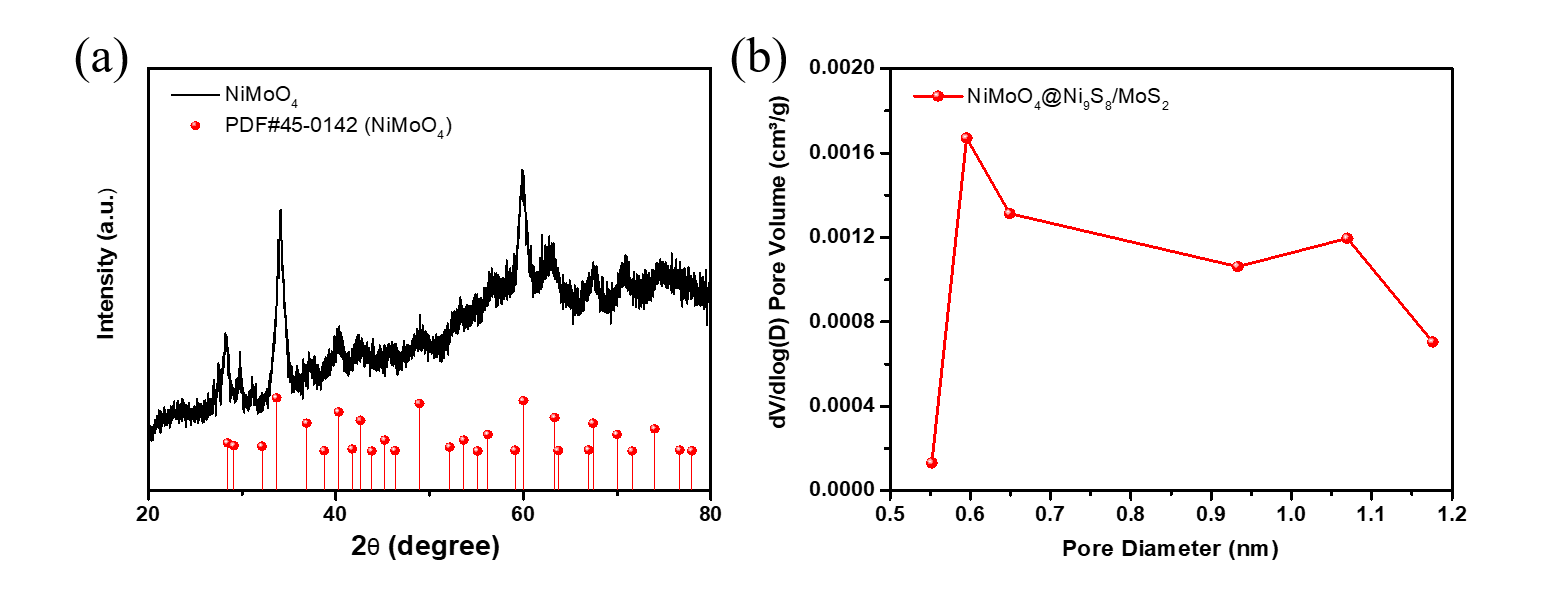
**

**Figure S2.** (a) XRD pattern and Rietveld refinement of NiMoO_4_; (b) Micropore size distribution profile of NiMoO_4_@Ni_9_S_8_/MoS_2_


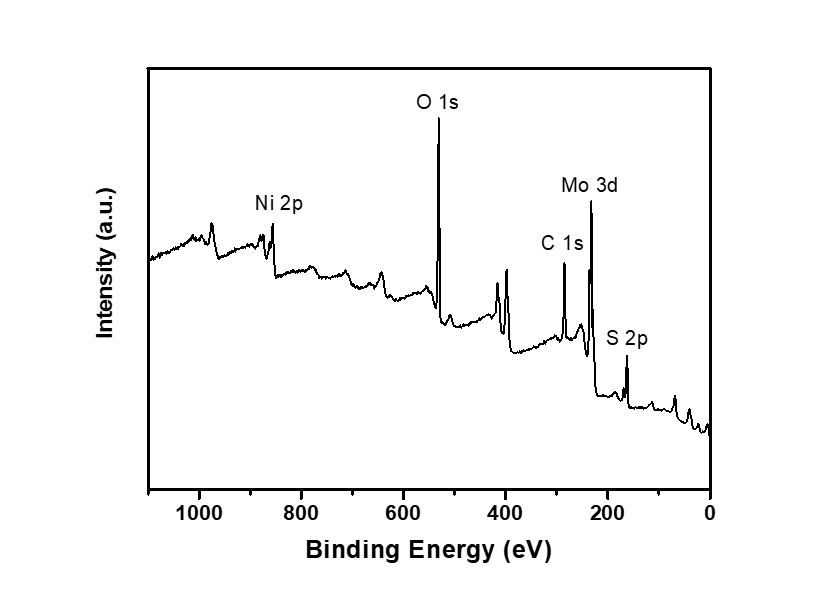


**Figure S3.** XPS survey spectrum, and high resolution XPS spectrum of NiMoO_4_@Ni_9_S_8_/MoS_2_.

**
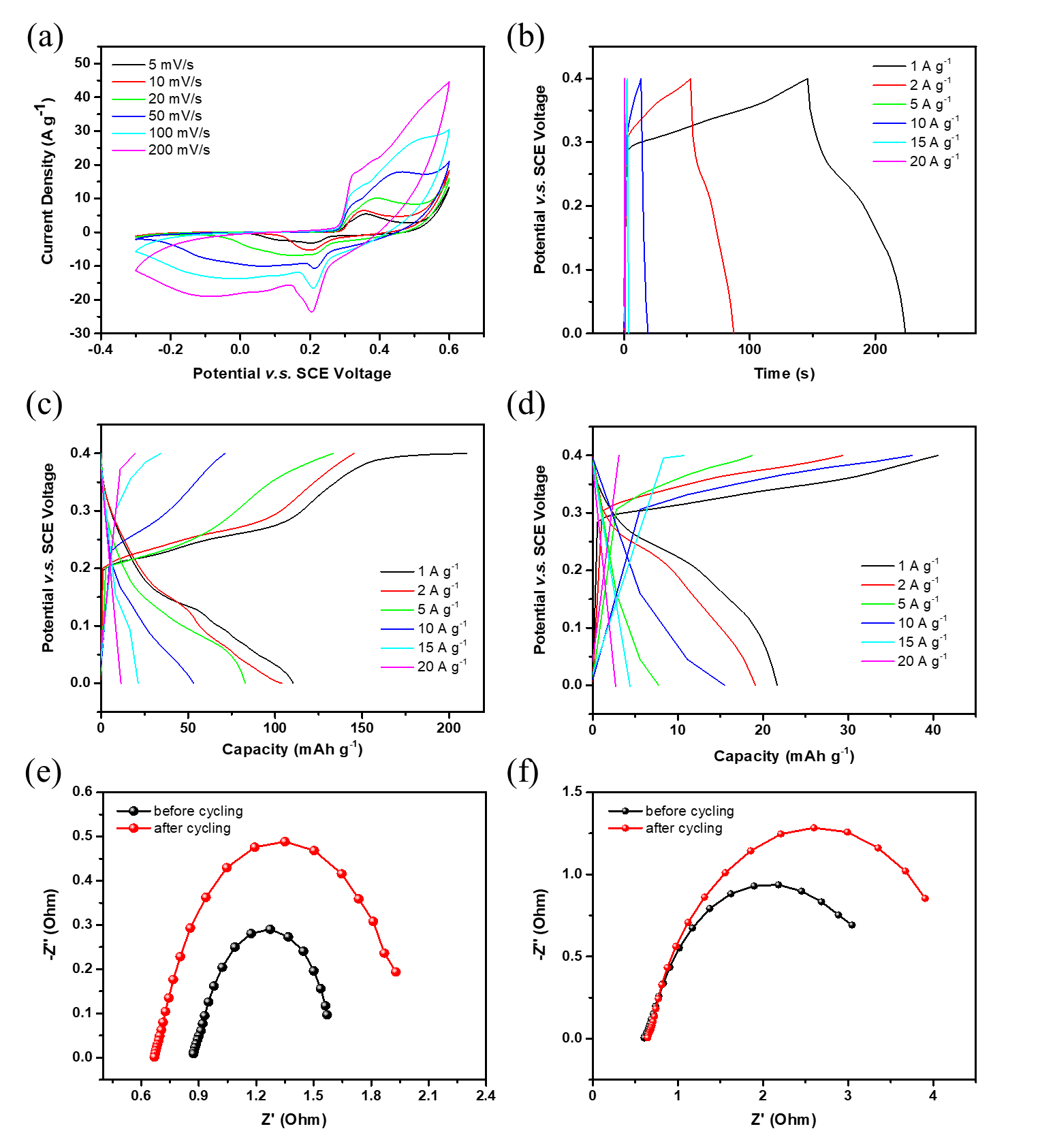
**

**Figure S4**. (a) CV curves of NiMoO_4_ at different scan rates; (b) GCD curves of NiMoO_4_ at various current densities; Charge-discharge profiles of (c) NiMoO_4_@Ni_9_S_8_/MoS_2_ and (d) NiMoO_4_ at different current densities; Nyquist plots of (e) NiMoO_4_@Ni_9_S_8_/MoS_2_ and (f) NiMoO_4_ before and after cycling.

**Table S1** Comparison of the performances of the our works and other reported works in alkline electrolyte

| Electrode material | Electrolyte | Specific capacitance at the given current density | Cycle number and  retention | Ref |
| --- | --- | --- | --- | --- |
| NiMoO_4_@Ni_9_S_8_/MoS_2_ nanorods | 6 M KOH | 373.4 F g^-1^ at 10 A g^-1^ | 81.0 %, 10,000 cycles at 10 A g^-1^ | Our work |
| C@MoS_2_/Ni_3_S_4_ | 2M KOH | 604.4 F g^-1^ (10 A g^-1^) | 80.1%, 10,000 cycles at 20 A g^-1^ | [1] |
| (Ni,Mo)S_2_/G composite | 2M KOH | 2379 F g^-1^ at 1 A g^-1^ | 60.7%, 10,000 cycles at 100 A g^-1^ | [2] |
| NiS_2_@MoS_2_ | 6 M KOH | 848.2 C g^-1^ at 1 A g^-1^ | 83.1%, 10,000 cycles at 10 A g^-1^ | [3] |
| Ni2Mo1 | 3 M KOH | 169.44 mAh g^-1^ at 1 A g^-1^ | 88.24%, 8000 cycles at 10 A g^-1^ | [4] |
| NiMoO_4_@MoS_2_ | 6 M KOH | 2246.7 F g^-1^ at 1 A g^-1^ | 88.4%, 5000 cycles at 5 A g^-1^ | [5] |
| NiS_2_ rods | 6 M KOH | 1020.2 F g^-1^ at 1 A g^-1^ | 84.1%, 4000 cycles at 2 A g^-1^ | [6] |
| NMS/CNT | 3 M KOH | 108 F g^-1^ at 0.5 A g^-1^ | 100%, 10,000 cycles at 5 A g^-1^ | [7] |
| 1-D NiMoO_4_ nanostructures | 1.0 M KOH | 1335 F g^-1^ at 1 A g^-1^ | 80.1%, 3000 cycles at 5 A g^-1^ | [8] |
| NiMoO_4_ nanostructure | 6 M KOH | 1475 F g^-1^ at 1 A g^-1^ | 87.9%, 5000 cycles at 20 A g^-1^ | [9] |
| NiMoO_4_/Ni_3_S_2_ nanohybrid | 3 M KOH | 1195.7 C g^-1^ at 0.5 mA cm^-2^ | 97.3%, 10,000 cycles, at 5 mA cm^-2^ | [10] |

**Reference**

[1] S. Qin, T. Yao, X. Guo, Q. Chen, D. Liu, Q. Liu, Y. Li, J. Li, D. He, MoS_2_/Ni_3_S_4_ composite nanosheets on interconnected carbon shells as an excellent supercapacitor electrode architecture for long term cycling at high current densities. Appl. Surf. Sci. **440**, 741-747 (2018)

[2] X. Yang, J. Mao, H. Niu, Q. Wang, K. Zhu, K. Ye, G. Wang, D. Cao, J. Yan, NiS_2_/MoS_2_ mixed phases with abundant active edge sites induced by sulfidation and graphene introduction towards high-rate supercapacitors. Chem. Eng. J. **406**, 126713 (2021)

[3] S. Hou, Y. Lian, Z. Xu, D. Wang, C. Ban, J. Zhao, H. Zhang, Construction of ball-flower like NiS_2_@MoS_2_ composite for high performance supercapacitors. Electrochim. Acta **330**, 135208 (2020)

[4] N. Wang, Q. Pan, X. Yang, H. Zhu, G. Ding, Z. Jia, Y. Wu, L. Zhao, High performance asymmetric supercapacitor based on Ni_x_S_y_/MoS_2_ nanoparticles. ACS Appl. Nano Mater. **2**, 4910-4920 (2019)

[5] L. Wan, J. Liu, X. Li, Y. Zhang, J. Chen, C. Du, M. Xie, Fabrication of core-shell NiMoO_4_@MoS_2_ nanorods for high-performance asymmetric hybrid supercapacitors. Int. J. Hydrog. Energy **45**, 4521-4533 (2020)

[6] Y. Ruan, J. Jiang, H. Wan, X. Ji, L. Miao, L. Peng, B. Zhang, L. Lv, J. Liu, Rapid self-assembly of porous square rod-like nickel persulfide via a facile solution method for high-performance supercapacitors. J. Power Sources **301**, 122-130 (2016)

[7] X. Yang, L. Zhao, J. Lian, Arrays of hierarchical nickel sulfides/MoS_2_ nanosheets supported on carbon nanotubes backbone as advanced anode materials for asymmetric supercapacitor. J. Power Sources **343**, 373-382 (2017)

[8] J. Hong, Y. W. Lee, B. Hou, W. Ko, J. Lee, S. Pak, J. Hong, S. M. Morris, S. Cha, J. I. Sohn, J. M. Kim, Solubility-dependent NiMoO_4_ nanoarchitectures: Direct correlation between rationally designed structure and electrochemical pseudokinetics. ACS Appl Mater Interfaces **8**, 35227-35234 (2016)

[9] V. S. Kumbhar, V. Q. Nguyen, Y. R. Lee, C. D. Lokhande, D.H. Kim, J.J. Shim, Electrochemically growth-controlled honeycomb-like NiMoO_4_ nanoporous network on nickel foam and its applications in all-solid-state asymmetric supercapacitors. New J. Chem. **42**, 14805-14816 (2018)

[10] L. Zhang, D. Zheng, S. Pei, L. Ye, S. Geng, J. Lian, Rational fabrication of nanosheet-dewy NiMoO_4_/Ni_3_S_2_ nanohybrid for efficient hybrid supercapacitor. J. Alloys Compd. **783**, 399-408 (2019)
